# mHealth Apps as Effective Persuasive Health Technology: Contextualizing the “Necessary” Functionalities

**DOI:** 10.2196/19302

**Published:** 2020-07-15

**Authors:** Allen McLean

**Affiliations:** 1 University of Saskatchewan Saskatoon, SK Canada

**Keywords:** persuasive health technology, behavioral intervention, persuasive systems design, eHealth, mHealth, nursing informatics

## Abstract

Persuasive health technology (PHT) is any technology purposely designed to influence, reinforce, change, or shape health-related attitudes or behaviors. Behavioral interventions can be developed for the purpose of maintaining or improving a person’s health status. Delivering behavioral interventions via PHTs is a promising approach for encouraging healthy behaviors among individuals and populations. Important attributes of all PHTs include their functionalities. A functionality refers to any useful features, functions, capabilities, or technologies associated with computer hardware or software. Creating effective PHTs requires a deliberate selection of appropriate functionalities for supporting specific behavioral interventions. The number and types of functionalities necessary to create an effective PHT will be specific to the context of each project, influenced by project objectives, stakeholder goals, behavioral interventions, and a variety of real-world constraints. Selecting appropriate functionalities can be challenging. Fortunately, there are frameworks and models developed specifically for guiding the design of PHTs. The Persuasive Systems Design model describes 4 categories, and 28 design principles for creating effective persuasive interventions. These same design principles could also be useful for guiding the selection of appropriate functionalities.

## Introduction

### Health Determinants

Numerous entwined factors influence a person’s individual health status, collectively known as the determinants of health. Some of these determinants are modifiable, while others are not. Organizations such as Health Canada [1] and the World Health Organization [2] include a person’s individual behaviors as a determinant of that person’s current health state, and future health trajectory. These behaviors include important decisions commonly made about, among others, diet, exercise, sexual activity, substance use, medication adherence, and vaccination [3].

Importantly, many individual health behaviors are modifiable, meaning that well-crafted behavioral interventions can be developed for the purpose of maintaining or improving a person’s individual health status. Behavioral health interventions are expected to remain important strategies for promoting health and well-being, preventing illness, and managing disease well into the foreseeable future [4,5]. Alongside traditional approaches (eg, individual coaching, motivational interviewing), delivering behavioral interventions via *persuasive health technologies* (ie, PHTs, such as mobile health [mHealth] technology) is a promising newer approach for encouraging healthy behaviors among individuals and populations [5-7]. However, these objectives can only be achieved if the designers and developers of these interventions and technologies select functionalities that provide *effective* support for achieving their goals.

### Objectives

This paper will describe the constellation of functionalities (eg, features, technologies) required for developing an effective mHealth-based PHT. Useful frameworks for designing PHTs will be introduced. A detailed list of current mobile IT functionalities will be presented, along with an example demonstrating how nurse researchers might design an effective PHT by matching various mHealth functionalities with a selected design framework and a behavioral intervention encouraging social/physical distancing. Finally, examples suggesting how 2 currently available mHealth systems might be adapted to serve as effective PHTs will be discussed.

### Background

Persuasive technology is any technology (eg, computers, websites, smartphones and apps, tablets, wearables, computer games) purposely designed to change attitudes or behaviors [8]. PHT (eg, electronic health [eHealth] programs, mHealth apps) is a specialized co-discipline that focuses on influencing, reinforcing, changing, or shaping health-related attitudes or behaviors [9] without using coercion or deception [10], and is most often used for health promotion and prevention or disease management [11]. PHTs for disease management help people comply with and adhere to treatment directives, such as better medication adherence or diabetes management [12]. The study of PHTs includes the design, research, ethics, and analysis of these interactive computing products [13]. The use of PHTs is growing rapidly in many areas of health and wellness [9]. Examples include PHTs encouraging behavior change in physical activity, healthy eating, tobacco cessation, risky sexual behavior, pregnancy, and dental health [12]. Peer-reviewed research has demonstrated benefits from the use of PHTs for promotion, prevention, and management across some chronic diseases [14-17], but there is tremendous opportunity for further study [12].

The WHO Global Observatory for eHealth [18] defines mHealth as medical and public health practice supported by mobile devices, such as smartphones, patient monitoring devices, personal digital assistants, and other wireless devices. Experts in the field of behavioral science agree that influencing healthy behavior change is notoriously difficult [3,19]. While there is increasing interest from researchers and clinicians in harnessing mHealth as a means of delivering behavioral interventions, academic research on the development and evaluation of effective health-related mHealth behavioral interventions is still in the early stages of study. Nevertheless, recent peer-reviewed studies and systematic reviews support (to varying degrees) the use of behavioral interventions delivered by mHealth across many populations and problems. Examples include sexual health [20], cardiovascular disease [21], diabetes [22-30], adolescents and young adults [31], ecological momentary assessments [32], health technology assessment [33], chronic disease management [34-36], sedentary behavior [37], diet and physical activity [38], and diabetic foot ulcers [39-41].

As a research tool, some mHealth solutions offer real-time and real-world measurements of phenomena. Some researchers ask study participants to maintain paper-and-pencil logs. However, the labor-intensive and intrusive nature of paper-and-pencil recording has limited its clinical applicability and underscores the concern that data can be backfilled by patients [42]. Smartphones offer a potential solution by enabling research through apps designed to prompt, collect, time stamp, and securely transfer patient data. Furthermore, researchers believe that conducting studies on smartphones is important because data collection is private, and may reduce potential bias from the Hawthorne effect.

Creating these technologies can be a demanding, but wonderfully creative and beneficial endeavor—developing solutions that combine knowledge from the health sciences, behavioral psychology, and software engineering for tackling many of the health problems experienced today. Each mHealth-based PHT will be made-to-measure, and the design and development of each behavioral intervention and technology will depend on a variety of factors, including project goals, health problem(s), mix of stakeholders (and their individual, possibly conflicting aspirations), advice received from consultation with subject-matter experts, input and feedback from users, available technologies, financial constraints, ethical considerations, and available *functionalities* [43,44].

For the purposes of this paper, a functionality refers to any useful technologies, capabilities, features, or functions associated with computer software or hardware, in particular mobile electronic devices (eg, smartphones, tablets, wearable devices) [45]. It is important to understand that functionalities are chosen to *support* a behavioral intervention [46], and intervention development and functionality selection should proceed in an iterative manner, each informing the other [6]. Furthermore, it is important to understand that the number and types of functionalities necessary to create an effective PHT will be specific to each mHealth project, that is, context will determine which functionalities are *necessary,* not algorithms or formulae [43]. For example, Dobson et al [47] conducted a randomized controlled trial investigating the effectiveness of a smartphone-based behavioral health intervention for supporting self-management among adults living with poorly controlled diabetes. The intervention group received a tailored package of SMS (short message service) text messages, in addition to usual care. The SMS text messages provided information, support, motivation, and reminders related to diabetes self-management and lifestyle behaviors. These researchers relied on a single mHealth functionality and technology (ie, SMS text messaging) and smartphones to deliver their intervention and answer their research and clinical questions. Notably, even when using these few functionalities, the intervention group demonstrated a significant reduction in HbA^1c^ (hemoglobin A^1c^; blood glucose), and a significant improvement in foot care behaviors, at 9 months’ follow-up.

A more complex PHT was proposed by Hussain et al [48], a personalized behavioral intervention for promoting awareness of optimal sun intake (ensuring sufficient vitamin D production), balanced with promoting safe sun exposure. Their persuasive health solution uses both smartphone and wearable technologies, together with multiple functionalities for achieving their project and clinical goals. Their mHealth app collects current weather, global positioning system (GPS), light exposure, and UV data from user’s smartphones and sensors in the wearable device. This information is then fed into a custom-built smartphone app providing personalized information to each user by tracking cumulative sun exposure and triggering appropriate smartphone alerts and UVI (UV index) warnings via SMS text messaging [49].

Creating effective PHTs requires a deliberate selection of appropriate functionalities for supporting specific behavioral interventions. In many ways, the process for developing an effective mHealth-based persuasive technology is similar to the development process followed for any other well-organized, well-executed software or technology development project [6,43,44], and correctly selecting which functionalities are needed is an important step in the development process [50]. However, even when following a recommended (eg, Agile, Scrum) software engineering methodology [6,51], this process is challenging, and choosing the appropriate functionalities that will result in an effective mHealth-based technology is not always so straightforward. Each PHT will be fit-for-purpose, meaning that cookbook solutions—such as if population X is experiencing health problem Y, then use functionalities a, b, and c to support behavioral intervention Z—do not exist. Confusing matters further, a functionality deemed useful in one context may not be in another. Fortunately, designers and developers can look to a small number of conceptual frameworks and models developed specifically for guiding the design of PHTs [52].

## Frameworks

Once a behavioral health intervention has been developed, and the decision made to deliver the intervention using mHealth technology, the intervention must be operationalized via the selected mobile IT functionalities (eg, SMS, GPS, sensors, cameras). A number of frameworks and models have proven useful for developing PHTs [10,52-54]. Among these, the *Persuasive Systems Design* (PSD) model is well-respected, and considered a state-of-the-art framework for designing and evaluating persuasive systems [9]. Many systematic reviews report limited evidence for the efficacy of mHealth apps [55], but note that projects developed using a conceptual framework or behavior theory are often more effective [5,51].

The PSD model is grounded in multiple theoretical constructs and established persuasive design techniques, with a focus on supporting the transfer of design specifications into software functionalities [56]. The PSD model has been applied widely across many disciplines, with researchers actively evaluating its use in many domains [17,57-62]. The PSD framework describes 28 persuasive design principles (Textbox 1) in 4 categories: primary task support, computer–human dialogue support, system credibility support, and social support [9,10]. The design principles found in the *primary task support* category support people when carrying out a primary task (eg, supporting regular blood glucose monitoring for people living with diabetes). Any interactive system provides some degree of system feedback to its users, and there are multiple design principles related to *dialogue support* that help users keep moving toward their goal or target behavior. The principles located in the *system credibility* category not surprisingly describe various ways to design a persuasive system with greater credibility. Lastly, the design principles found in the *social support* category describe how to motivate users by leveraging social influence using concepts such as competition or cooperation [9,10,56].

**Persuasive Systems Design framework: design principles [10].**

**1. Primary task support**

- Reduction: A system that reduces complex behavior into simple tasks helps users perform the target behavior, and it may increase the benefit-to-cost ratio of a behavior.
- Tunneling: Using the system to guide users through a process or experience provides opportunities to persuade along the way.
- Tailoring: Information provided by a system is more persuasive if it is tailored to the needs, interests, personality, usage context, or other factors relevant to a user group.
- Personalization: A system that offers personalized content or services has a greater capability for persuasion.
- Self-monitoring: A system that keeps track of one’s own performance or status supports the user in achieving goals.
- Simulation: A system that provide simulations can persuade by enabling users to observe immediately the link between cause and effect.
- Rehearsal: A system providing a means by which to rehearse a behavior that can enable people to change their attitudes or behavior in the real world.

**2. Computer–human dialogue support**

- Praise: By offering praise, a system can make users more open to persuasion.
- Rewards: Systems that reward target behaviors may have greater persuasive powers.
- Reminders: If a system reminds users of their target behavior, the users will more likely achieve their goals.
- Suggestion: Systems offering fitting suggestions will have greater persuasive powers.
- Similarity: People are more readily persuaded through systems that remind them of themselves in some meaningful way.
- Liking: A system that is visually attractive for its users is likely to be more persuasive.
- Social role: If a system adopts a social role, users are more likely to use it for persuasive purposes.

**3. System credibility support**

- Trustworthiness: A system viewed as trustworthy will have increased powers of persuasion.
- Expertise: Incorporating expertise will have increased powers of persuasion.
- Surface credibility: System credibility is based on a firsthand inspection.
- Real-world feel: A system that highlights people or organization behind its content or services will have more credibility.
- Authority: A system leveraging roles of authority will be more persuasive.
- Third-party endorsements: Third-party endorsements, especially from well-known and respected sources, boost perceptions of system credibility.
- Verifiability: Credibility perceptions will be enhanced if a system makes it easy to verify the accuracy of site content via outside sources.

**4. Social support**

- Social learning: A person will be more motivated to perform a target behavior if he or she can use a system to observe others performing the behavior.
- Social comparison: System users will have a greater motivation to perform the target behavior if they can compare their performance with the performance of others.
- Normative influence: A system can leverage normative influence or peer pressure to increase the likelihood that a person will adopt a target behavior.
- Social facilitation: System users are more likely to perform target behavior if they discern via the system that others are performing the behavior along with them.
- Cooperation: A system can motivate users to adopt a target attitude or behavior by leveraging humans’ natural drive to co-operate.
- Competition: A system can motivate users to adopt a target attitude or behavior by leveraging humans’ natural drive to compete.
- Recognition: By offering public recognition for an individual or group, a system can increase the likelihood that a person/group will adopt a target behavior.

## Functionalities

Modern mobile devices provide many functionalities; even low-cost phones, tablets, and wearables (eg, smart watches, fitness trackers) can be useful as PHTs. Functionalities will vary by device, often influenced by cost, manufacturer, or hardware specifications, though many will share a number of common features and capabilities. Important hardware functionalities include the display (usually touchscreen), keyboard, headphone jack, camera, volume controls, water-resistant capability, and rechargeable battery. Important communication functionalities include calling, SMS text messaging, multimedia messaging service (MMS; audio and video playback), web browser, email, and apps [63,64]. An incredible amount of sensor data can be collected, including location data (GPS, latitude/longitude, altitude, speed, and bearing), motion data (accelerometer, gravity, gyroscope, magnetic field, and pedometer), environmental data (ambient temperature, light, pressure, proximity, and relative humidity), network data (Bluetooth and Bluetooth networks), and others including battery usage, screen state, app usage, and ambient audio [65]. Wearable devices can also collect a variety of physiological data such as heart rate, sleep tracking, electrocardiogram, steps, and calories burned, to name a few [66,67].

It is meaningless to think of each functionality, or combination of functionalities, as effective or ineffective in the context of designing or developing a PHT. An effective mHealth-based solution is the result of selecting appropriate functionalities supporting a specific behavioral intervention. Linking functionalities to the PSD model can assist with this process.

## Example

Imagine a team of nurse researchers planning the design for an effective PHT delivered via mHealth technology. To achieve this goal, these nurses must create a behavioral intervention, then select a design framework, and choose functionalities that can operationalize their intervention. The PSD framework describes 28 persuasive design principles (Textbox 1) in 4 categories; these same design principles are useful for guiding the selection of appropriate functionalities. Textbox 2 illustrates how an imagined behavioral intervention (encouraging social/physical distancing) suggested during a global pandemic (eg, COVID-19) could be developed by linking design principles from the PSD model with various context-appropriate mHealth functionalities. It is important to note that not every design principle will be useful for selecting functionalities that support a behavioral intervention; this will be project specific, and limited by a variety of real-world constraints such as budgets or technologies.

**Examples linking design principles with functionalities for the purpose of developing a PHT for encouraging social/physical distancing.**

**1. Primary task support**

- Reduction: A system that reduces complex behavior into simple tasks helps users perform the target behavior, and it may increase the benefit-to-cost ratio of a behavior.
*For example, using SMS text or MMS messages, or the ability to link to instructional web-based videos for delivering social/physical distancing instructions*
- Tailoring: Information provided by a system is more persuasive if it is tailored to the needs, interests, personality, usage context, or other factors relevant to a user group.
*For example, using feedback from individual user surveys, alerts, Bluetooth data, or sensor data that provides personalized social/physical distancing information important for each individual user and delivered via SMS text or MMS messaging*
- Self-monitoring: A system that keeps track of one’s own performance or status supports the user in achieving goals.
*For example, feedback and information provided to users about their daily social/physical distancing habits directly from individual sensor data, Bluetooth data, or health and wellness tracking apps*

**2. Computer–human dialogue support**

- Praise: By offering praise, a system can make users more open to persuasion.
*For example, praise delivered by automated SMS text or MMS messages, triggered by survey responses, or physiologic and proximity sensor data when proper social/physical distancing practices are recorded*
- Reminders: If a system reminds users of their target behavior, the users will more likely achieve their goals.
*For example, alerts and reminder messages about effective social/physical distancing delivered via mobile devices or wearables and triggered by, for example, time of day, GPS location, motion sensors, or proximity sensors*
- Suggestion: Systems offering fitting suggestions will have greater persuasive powers.
*For example, suggestions for improving the effectiveness of social/physical distancing practices delivered via SMS text or MMS messages and triggered by, for instance, time of day, GPS location, Bluetooth data, motion or proximity sensors*

**3. System credibility support**

- Surface credibility: System credibility is based on a firsthand inspection.
*For example, building systems using professional user experience software design*
- Authority: A system leveraging roles of authority will be more persuasive.
*For example, including content from local, national, or international health agencies and their social/physical distancing recommendations*
- Third-party endorsements: Third-party endorsements, especially from well-known and respected sources, boost perceptions of system credibility.
*For example, including SMS text messages from family members, friends, or celebrities*

**4. Social support**

- Social facilitation: System users are more likely to perform target behavior if they discern via the system that others are performing the behavior along with them.
*For example, sharing user data from health or wellness tracking apps, individual sensor data, or survey responses via SMS text or MMS messaging may also facilitate cooperation*
- Competition: A system can motivate users to adopt a target attitude or behavior by leveraging humans’ natural drive to compete.
*For example, sharing user data from health or wellness tracking apps, individual sensor data, or survey responses via SMS text or MMS messaging, or using a bespoke app that tracks social/physical distancing data*
- Recognition: By offering public recognition for an individual or group, a system can increase the likelihood that a person/group will adopt a target behavior.
*For example, sharing user or group progress with other users about social/physical distancing*

## Conclusions

Creating effective mHealth-based PHTs requires a deliberate selection of necessary functionalities. These functionalities are only *necessary* in the context of providing support for specific behavioral interventions. To operationalize a behavioral intervention, nurse researchers can build a bespoke PHT themselves (assuming they have the required technical skills), hire professional designers and developers (assuming they have significant financial resources), or modify an existing platform (assuming such platforms exist, and permission to modify is possible at an affordable cost).

This third option is appealing because it can be quick to develop, affordable, and requires limited technical skills. For example, the Ethica research platform provides researchers with many functionalities [68] and could be adapted for use as a persuasive technology. When comparing Ethica functionalities with the PSD model design principles, it appears Ethica could offer support for every principle, meaning that Ethica could offer support for any behavioral intervention developed using the PSD model. By contrast, the REDCap research platform provides researchers with far fewer functionalities [69], mainly lacking the ability to process sensor data, but it could also be adapted for use as a persuasive technology. This means REDCap could offer support for a smaller number of PSD model design principles and behavioral interventions. Does this mean REDCap is less useful than Ethica as a PHT? Not necessarily; as argued in this paper, context and requirements will determine the *necessary* functionalities.

## Abbreviations

MMS: multimedia messaging service
PHT: persuasive health technology
SMS: short message service
WHO: World Health Organization
